# Supplementary material for: CNN-based classifier for automated identification of magnetic states in spin dynamics simulations
Source: Sci Rep. 2026 Jun 1;16:16834. doi: 10.1038/s41598-026-54755-y (PMC13226725; doi:10.1038/s41598-026-54755-y)
Supplement: Supplementary file 1 — Supplementary Information. [file 41598_2026_54755_MOESM1_ESM.pdf]

Supplementary Materials

CNN-Based Classifier for Automated Identification of Magnetic States in Spin Dynamics Simulations

Amal Aldarawsheh, Ahmed Alia and Stefan Blügel

Supplementary Table 1: Representative Hamiltonian parameter (in meV) intervals used during dataset generation across the considered lattice types.

State	Cell type	J_1	J_2	J_3	K	B	DMI
AFM	Triangular	-1	-1	1	[-1,1]	0	0
	Rectangular	-1	[-1,1]	0	[-0.3,0.3]	0	0
	Square	-1	[-2,1]	0	[-0.3,0.3]	0	0
	Rhombic	-1	0	0	[-1,1]	0	0
FM	Triangular	1	0	0	[-1,1]	0	0
	Rectangular	1	0	0	[-0.5,0.5]	0	0
	Square	1	0	0	[-0.5,0.5]	0	0
	Rhombic	1	0	0	[-0.5,0.5]	0	0
AFM skyrmions	Triangular	-1	[-0.4,0.2]	[0.05,0.15]	[0.01,0.03]	0	[0.04,0.1]
	Rectangular	-1	-1	[-5,0.5]	[0.001,0.4]	0	[0.2,0.7]
	Square	-1	-2	0	[0.01,0.2]	0	[0.1,0.3]
	Rhombic	-1	0	0	[0.015,0.6]	0	[0.1,0.4]
AFM in-plane skyrmions	Triangular	-1	[-0.4,-0.1]	[0.05,0.15]	[-0.2,-0.04]	0	[0.04,0.1]
	Rectangular	-1	-1	[-2,-5]	[-0.08,-0.3]	0	[0.2,0.5]
	Square	-1	-2	0	[-0.07,-0.3]	0	[0.2,0.5]
	Rhombic	-1	0	0	[-0.5,-0.035]	0	[0.1,0.4]
FM skyrmions	Triangular	1	0	0	[0.01,0.6]	[0,0.5]	[0.1,0.5]
	Rectangular	1	1	0	[0.01,0.6]	[0.01,0.5]	[0.1,0.5]
	Square	1	0	0	[0.01,0.6]	[0,0.5]	[0.1,0.5]
	Rhombic	1	0	0	[0.1,0.3]	[0.0,2.5]	[0.1,0.3]
FM in-plane skyrmions	Triangular	1	0	0	[-0.8,-0.1]	0	[0.2,0.9]
	Rectangular	1	[1,2]	0	[-0.03,-0.4]	0	[0.1,0.4]
	Square	1	0	0	[-0.05,-0.5]	0	[0.1,0.4]
	Rhombic	1	0	0	[-0.05,-1]	0	[0.1,0.4]
AFM stripe domains	Triangular	-1	[-0.2,0]	[0.01,0.1]	[0.001,0.1]	0	[0.03,0.1]
	Rectangular	-1	-1	0	0	[0,0.3]	[0.1,0.6]
	Square	-1	0	0	0	[0,0.3]	[0.1,0.6]
	Rhombic	-1	0	0	[0,0.1]	0	[0.4,0.7]
FM stripe domains	Triangular	1	0	0	0	[0.0,0.5]	[0.2,0.9]
	Rectangular	1	[1,4]	0	[0,0.1]	[0.0,0.4]	[0.1,0.6]
	Square	1	[2,4]	0	[0.0,0.3]	[0.0,0.4]	[0.2,0.5]
	Rhombic	1	0	0	[0,0.4]	[0,5]	[0.4,0.7]
Néel	Triangular	-1	[0,1.5]	[-1.5,0]	[-1,1]	0	0

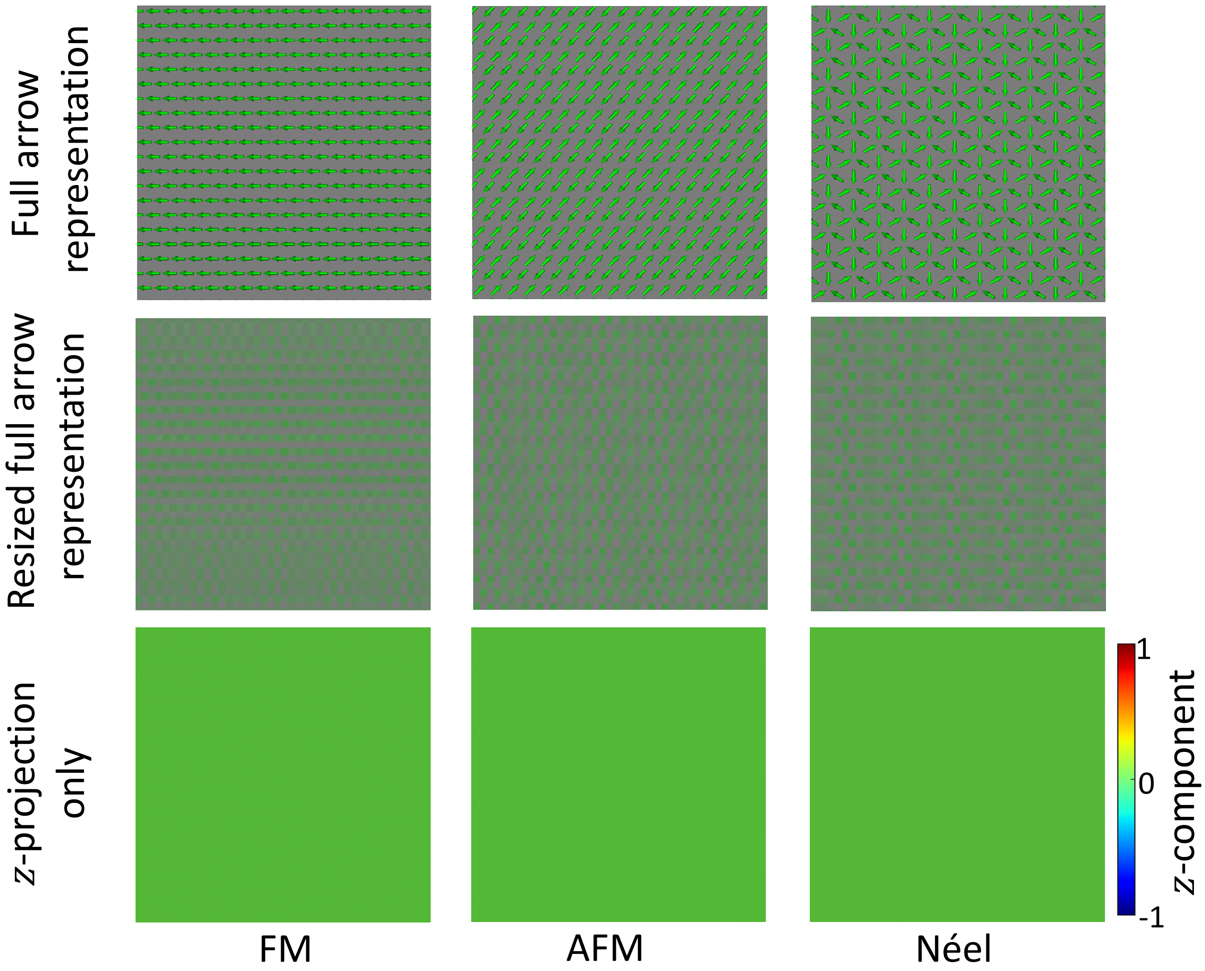

Supplementary Figure 1: Comparison of representative cropped regions of in-plane FM, AFM, and Néel states rendered in three forms: the original full-arrow rendering, the resized full-arrow rendering used as CNN input, and the corresponding z-projection-only visualization. The full-arrow representation preserves the in-plane directional structure after resizing, whereas the z-projection-only representation removes class-relevant information needed to distinguish the in-plane cases.

Supplementary Table 2: Quantitative comparison of representative in-plane FM, AFM, and Néel states for the full-arrow and z-projection-only representations using the structural similarity index measure (SSIM), cosine similarity, and Euclidean distance. Higher SSIM and cosine similarity indicate greater similarity, whereas lower Euclidean distance indicates smaller differences between images.

Representation	Class pair	SSIM	Cosine similarity	Euclidean distance
Full-arrow	FM vs AFM	0.0007	0.6555	8.4125
Full-arrow	FM vs Néel	0.3811	0.8148	8.5513
Full-arrow	AFM vs Néel	0.4800	0.7740	6.5873
z-projection-only	FM vs AFM	1.0000	1.0000	0.0000
z-projection-only	FM vs Néel	1.0000	1.0000	0.0000
z-projection-only	AFM vs Néel	1.0000	1.0000	0.0000
